# Effects of synbiotic supplementation on intestinal microbiota composition in children and adolescents with exogenous obesity: (Probesity-2 trial)

**DOI:** 10.1186/s13099-023-00563-y

**Published:** 2023-07-21

**Authors:** Gonca Kilic Yildirim, Meltem Dinleyici, Yvan Vandenplas, Ener Cagri Dinleyici

**Affiliations:** 1 Faculty of Medicine, Pediatrics Nutrition and Metabolism Unit, Eskisehir Osmangazi University, Eskisehir, Turkey; 2 Faculty of Medicine, Department of Social Pediatrics, Eskisehir Osmangazi University, Eskisehir, Turkey; 3 Vrije Unversiteit Brussel, UZ Brussel, KidZ Health Castle, Brussels, Belgium; 4 Faculty of Medicine, Department of Pediatrics, Eskisehir Osmangazi University, Eskisehir, TR-26040 Turkey

**Keywords:** Obesity, Children, Adolescent, Probiotic, Synbiotic, Microbiota

## Abstract

**Introduction:**

Gut microbiota manipulation may be a potential therapeutic target to reduce host energy storage. There is limited information about the effects of probiotics/synbiotics on intestinal microbiota composition in children and adolescents with obesity. The objective of this randomized double-blind placebo-controlled trial was to test the effects of a multispecies synbiotic on intestinal microbiota composition in children and adolescents with exogenous obesity.

**Method:**

Children with exogenous obesity were managed with a standard diet and increased physical activity and were randomly allocated into two groups at a ratio of 1:1; the 1st group received synbiotic supplementation (probiotic mixture including *Lactobacillus acidophilus, Lacticaseibacillus. rhamnosus, Bifidobacterium bifidum, Bifidobacterium longum, Enterococcus faecium* (total 2.5 × 10^9^ CFU/sachet) and fructo-oligosaccharides (FOS; 625 mg/sachet) for 12 weeks; the 2nd group received placebo once daily for 12 weeks. Fecal samples were obtained before and at the end of the 12-week intervention to characterize the changes in the gut microbiota composition. Detailed metagenomic and bioinformatics analyses were performed.

**Results:**

Before the intervention, there were no significant differences in alpha diversity indicators between the synbiotic and placebo groups. After 12 weeks of intervention, the observed taxonomic units and Chao 1 were lower in the synbiotic group than at baseline (p < 0.001 for both). No difference for alpha diversity indicators was observed in the placebo group between baseline and 12 weeks of intervention. At the phylum level, the intestinal microbiota composition of the study groups was similar at baseline. The major phyla in the synbiotic group were Firmicutes (66.7%) and Bacteroidetes (18.8%). In the synbiotic group, the Bacteroidetes phylum was higher after 12 weeks than at baseline (24.0% vs. 18.8%, p < 0.01). In the synbiotic group, the Firmicutes/Bacteroidetes ratio was 3.54 at baseline and 2.75 at 12 weeks of intervention (p < 0.05). In the placebo group, the Firmicutes/Bacteroidetes ratio was 4.70 at baseline and 3.54 at 12 weeks of intervention (p < 0.05). After 12 weeks of intervention, the Firmicutes/Bacteroidetes ratio was also lower in the synbiotic group than in the placebo group (p < 0.05). In the synbiotic group, compared with the baseline, we observed a statistically significant increase in the genera Prevotella (5.28–14.4%, p < 0.001) and Dialister (9.68–13.4%; p < 0.05). Compared to baseline, we observed a statistically significant increase in the genera Prevotella (6.4–12.4%, p < 0.01) and Oscillospira (4.95% vs. 5.70%, p < 0.001) in the placebo group. In the synbiotic group, at the end of the intervention, an increase in *Prevotella, Coprococcus, Lachnospiraceae* (at the genus level) and *Prevotella copri, Coprococcus eutactus, Ruminococcus spp.* at the species level compared to baseline (predominance of *Eubacterium dolichum, Lactobacillus ruminis, Clostridium ramosum, Bulleidia moorei*) was observed. At the end of the 12th week of the study, when the synbiotic and placebo groups were compared, *Bacteroides eggerthi* species were dominant in the placebo group, while *Collinsella stercoris* species were dominant in the synbiotic group.

**Conclusion:**

This study is the first pediatric obesity study to show that a synbiotic treatment is associated with both changes intestinal microbiota composition and decreases in BMI. Trial identifier: NCT05162209 (www.clinicaltrials.gov).

**Supplementary Information:**

The online version contains supplementary material available at 10.1186/s13099-023-00563-y.

## Significance

**What is Known**

- Gut microbiota manipulation may be a potential therapeutic target to reduce host energy storage.

- There are studies on the use of probiotics and prebiotics as a support for treatment in obesity and effects on microbiota composition, but most of these studies were conducted in adult age groups.

- Most of the studies on the effects of probiotics and synbiotics on obesity are related to anthropometric measurements, lipid parameters and non-alcoholic fatty liver disease, and there are few studies regarding their effects on intestinal microbiota composition, especially in children.

**What is New**

- This study is the first pediatric obesity study to show that a synbiotic use associated with changes intestinal microbiota composition.

- In the synbiotic group, at the end of the intervention, an increase in *Prevotella, Coprococcus, Lachnospiraceae* at the genus level and *Prevotella copri, Coprococcus eutactus, Ruminococcus spp.* at the species level compared to baseline (predominance of *Eubacterium dolichum, Lactobacillus ruminis, Clostridium ramosum, Bulleidia moorei*) was observed.

**Supplementary Information:**

The online version contains supplementary material available at 10.1186/s13099-023-00563-y.

## Introduction

The etiology of obesity is multifactorial, including genetic predisposition and environmental factors. In addition to these factors, the gut microbiota has been reported as a factor associated with overweight and obesity [1]. The microbiota consists of a diverse and complex community of organisms, including bacteria, viruses, bacteriophages, fungi and archaea, that together contribute essential functions for host metabolism and thereby impact health and disease states [2]. Microbiota have basic functions, such as digestion, maturation and development of the immune system, inhibition of adhesion of pathogenic microorganisms and gut-brain interaction. The gastrointestinal microbiota plays an important role in the synthesis and absorption of many nutrients and metabolites [3, 4]. It has been shown that the development of microbiota composition in children begins in the mother’s womb and is shaped in the first 1000 days of life. During pregnancy, it has been shown that the mother’s weight and body mass index (BMI), nutritional habits, weight gain, diseases during pregnancy, medications and the psychological state of the mother influence the mother-infant dyad microbiota composition. Mode of delivery, prematurity, birthweight, neonatal intensive care hospitalization, breastfeeding and perinatal antibiotic use are also main factors affecting microbiota. Along with puberty, hormonal changes, nutrition and obesity influence microbiota composition. Dietary habits and/or obesity relate to changes in the composition of the gut microbiota. Geography, diet, physiological variations and lifestyle changes affect microbiota composition [5–7].

Gut microbiota manipulation may be a potential therapeutic target to reduce host energy storage [8]. Although a causal relationship between gut microbiota, nutrition and obesity has not yet been established, current evidence suggests that probiotic, prebiotic, synbiotic or postbiotic supplements aiming to improve microbiota composition and diversity may have positive effects on gut health [9–13]. The International Scientific Association of Probiotics and Prebiotics (ISAPP) defines probiotics as “live microorganisms that have been shown to have positive effects on health when taken in adequate amounts” [9]. The International Scientific Association for Probiotics and Prebiotics (ISAPP) defined prebiotics as “*a substrate that is selectively utilized by host microorganisms conferring a health benefit*” and defined synbiotics as “*a mixture comprising live microorganisms and substrate(s) selectively utilized by host microorganisms that confers a health benefit on the host*” [10, 11].

The standard treatment of obesity in children is based on a reduction in energy intake by regulating the diet and increasing energy expenditure by increasing activity [14]. Dietary interventions with probiotics, prebiotics or synbiotics aimed at correcting disruption of the gut microbiota observed in obesity or following imbalanced diets may provide health benefits by facilitating weight loss and maintenance. It has been shown that there are changes in the composition of the microbiota, decreases in body weight and fat mass, improvements in lipid levels, fasting glucose and insulin levels, and decreases in inflammatory factors as a result of the intake of probiotics and prebiotics [15, 16]. There are studies on the use of probiotics and prebiotics as a support for treatment in obesity and effects on microbiota composition, but most of these studies were conducted in adult age groups. Studies on the effects of synbiotics on obesity in children are limited [17, 18].

We previously showed that taking a specific synbiotic for 12 weeks in addition to dietary and physical activity recommendations had a positive effect on anthropometric measurements, resulting in a 4% reduction in body weight, a 5.1% reduction in BMI, a 6% reduction in waist circumference, and a 2.4% reduction in hip circumference in a randomized placebo-controlled study [19]. To the best of our knowledge, no study has evaluated the effects of synbiotics on the intestinal microbiota composition in obese children. In this part of our study, we evaluated the intestinal microbiota composition of this study cohort.

## Patients and methods

### Study design

This is a single-center, prospective, randomized, double-blind, placebo-controlled clinical study in children aged between 8 and 17 years with exogenous obesity who presented for the first time to the Eskişehir Osmangazi University Faculty of Medicine, Department of Pediatrics, Nutrition and Metabolism, between January 2019 and June 2021 [19]. This clinical study was planned and performed in accordance with the Declaration of Helsinki and Good Clinical Practice guidelines, patient rights regulation and ethical committees. Permission for the study was obtained from the Clinical Research Ethics Committee of Eskişehir Osmangazi University Faculty of Medicine with Decision Number 54 on September 27, 2018. This study is registered in ClinicalTrials.gov under the Identifier number NCT05162209. Written informed consent was obtained from all parents and children prior to inclusion. Study results are shown according to Strengthening The Organization and Reporting of Microbiome Studies (STORMS) [20].

### Study Population, inclusion and exclusion criteria

Children and adolescents aged 8 to 17 years with a BMI equal to or higher than the age- and sex-specific 95th revised percentiles of the Centers for Disease Control and Prevention (CDC) were evaluated according to the study criteria [21]. Patients who had no pathological findings other than obesity in the physical examination, whose height was compatible or tall with the chronological age, and whose mentality was normal were considered exogenous obese and included in the study. Patients with secondary obesity or endogenous obesity, history of any chronic diseases and/or chronic medication use and/or monogenic syndromes and other genetic syndromes, or those under special diets, as well as patients with exogenous obesity with insulin resistance and hypertension were excluded from the study. Patients who used probiotics/synbiotics/fibers or antibiotics in the 8 weeks before the application date were excluded from the study (24). The flow chart of the study according to the STORMS guidelines is shown in Supplementary Fig. 1. Baseline and 12 weeks anthropometric measurements and laboratory findings were shown in Supplementary Table 1.

### Diet and increasing physical activity

The definition of obesity, its effects on the body, complications and how the treatment would be were explained in detail to the patients and their families for approximately 30 min. A dietary intervention and increased physical activity were recommended in all cases. The diets of the patients were reduced by 10% from their habitual intake; the daily cholesterol intake was regulated to not exceed 300 mg, with 30% of energy provided from fats, 15% from proteins and 55% from complex carbohydrates. In addition to their normal activities, the patients were advised to exercise moderately for at least 30 min daily.

### Randomization, intervention and masking

The patients were divided into two groups by a computer-generated randomization sequence that assigned participants in a 1:1 allocation ratio to treatment with synbiotics or placebo with blocks of 8, blinding the study team, patients and their relatives. Interventional products were numbered, and all investigators and patients were blinded for the duration of the study. The treatment duration was 12 weeks. In the first group, 1 sachet each day for 12 weeks (*Lactobacillus acidophilus* (4.3 × 10^8^ CFU/sachet), *Lacticaseibacillus rhamnosus* (4.3 × 10^8^ CFU/sachet), *Bifidobacterium bifidum* (4.3 × 10^8^ CFU/sachet), *Bifidobacterium longum* (4.3 × 10^8^ CFU/sachet), *Enterococcus faecium* (8.2 × 10^8^ CFU/sachet), total 2.5 × 10^9^ CFU per sachet, fructooligosaccharide (FOS) 625 mg, lactulose 400 mg, Vitamin A (6 mg), Vitamin B1 (1.8 mg), Vitamin B2 (1.6 mg), Vitamin B6 (2.4 mg), Vitamin E (30 mg), Vitamin C (75 mg) were given. The second study group was given a placebo (contain the same amounts of vitamins) consisting of a sachet with shape, taste, and smell identical to the synbiotic sachet for 12 weeks.

### Outcomes

The aim of this part was to evaluate the effects of 12 weeks of intake of a multistrain synbiotic on gut microbiota composition in children with exogenous obesity. We planned to evaluate alpha and beta diversity indices, amplicon sequence variants abundance, taxonomic ratios, comparison for significant taxonomies.

### Sample collections

Stool samples were obtained from participants at baseline and at the end of the intervention (end of the 12th week). Fresh tool samples (at least 5 ml) received at hospital, were collected in 50 cc Falcon tubes, frozen immediately, and stored upright at -80 °C without any treatment. All samples were delivered to the laboratory where DNA analysis was carried out in accordance with the cold chain rules every three months.

### Fecal DNA extraction, sequencing and bioinformatic analysis

The QuickGene (DNA extraction kit from tissue) extraction device was used for the DNA extraction protocol from stool samples. First, 25 mg of stool sample was transferred to a homogenization tube with 250 µl of MDT (tissue lysis) solution. To homogenize, 15 mg of 0.1 mmø glass beads or 10 1.0 mmø zirconia beads were added to the tube. Then, 2 × 120 s of application was made at 5000 rpm in the homogenizer (Thermo Scientific FastPrep FP120 Cell tissue Disrupter homogenizer, United States). After the sample was homogenized, 25 µl of EDT (Proteinase K) solution was added and incubated at 56 °C for 60 min. Then, it was centrifuged at 15,000 g for 10 min at room temperature. After centrifugation, 200 µl of supernatant was transferred to a 1.5 mL microtube. After 180 µl of LDT (Cell Lysis) solution was added and vortexed for 15 s, the microtube was incubated at 70 °C for 10 min. In the next step, 240 µl of 99% cold ethanol was added and vortexed for 15 s. The entire contents of the microtube were transferred to the QuickGene (Kurabo, Japan) filtered cassette, and washes and elutions were performed following the instrument protocol. Three washes were performed using 750 µl of WDT (wash buffer) solution. As a result of the extraction process, bacterial 16 S ribosomal RNA (rRNA) gene target sequencing was performed from the materials obtained in the study (https://support.illumina.com/documents/documentation/chemistry_documentation/16s/16s-metagenomic-library-prep-guide-15044223-b.pdf). The resulting genomic DNA was amplified with 16 S V3-V4 314 F-860R primer sets, and library preparation was performed with a Nextera XT DNA library preparation kit and indices (Illumina, CA, USA). Amplicon libraries were cleaned by selecting large fragments (AMPure XP, Beckman Coulter). It was then normalized and aggregated. After the library was prepared, the NovaSeq 6000 (Illumina, CA, USA) instrument was used to run the sequencing.

Pair-ended Illumina reads (2 × 250) were transferred to the QIIME2 environment [22]. All of the samples had sequence depths greater than 100X, and no samples were omitted from the run. Quality clipping, chimera detection, and cleaning of reads were implemented through the QIIME2 Dada2 pipeline (via q2-dada2) [23]. Amplicon sequence variants (ASVs) generated by Dada2 were mapped to the GreenGenes (http://greengenes.lbl.gov) database [24, 25]. The Phyloseq [25] object was created from QIIME2 artifact files in the R 4.1 environment [26, 27]. Alpha diversity metrics (Chao1 diversity, Shannon and Simpson index) were calculated from the phyloseq object with the microbiome R package. Significant differences between groups were calculated with the Kruskal-Wallis rank sum test. Beta diversity was computed with phyloseq, including Bray-Curtis, Jaccard, unweighted UniFrac and weighted UniFrac distance metrics. Beta diversity statistical significance between groups was calculated by the PERMANOVA test via the Adonis function from the vegan R package. Intergroup p values were calculated with the Kruskal-Wallis test. Specific differences between groups were determined by differential abundance analysis with the Deseq2 R package [28]. Linear discriminant analysis effect size (LEfSe) analysis was performed between groups to show statistically significant taxonomies [29].

## Results

Bioinformatic analysis was performed in 28 children in the synbiotic group and 26 children in the placebo group. Alpha diversity (within-sample species diversity) was evaluated with Chao1 (a measure of community richness), observed ASVs, Shannon (a measure of richness and evenness or entropy) and Simpson indices, which were used to measure species richness and evenness (similar abundance) in the groups. While there was no difference in the Shannon (which measures richness) and Simpson indices (data not shown) in the synbiotic group at the beginning and at the end of the 12th week, the observed ASVs and Chao1 indices were found to be lower at the 12th weeks compared to the initial period (p < 0.001) (Supplementary Fig. 3). There was no difference between theASVs, Chao-1, Simpson (data not shown), and Shannon indices observed at the beginning of the placebo group and at the end of the 12th week (p > 0.05) (Supplementary Figs. 2 and 3). At the 12 weeks of intervention, the Chao1 index was found to be lower in the synbiotic group than in the placebo group, but there was no significant difference (Supplementary Fig. 2). Bray–Curtis dissimilarity was used to compare the abundance of each ASV between the synbiotic and placebo groups. The β-diversity (between-sample dissimilarity) weighted UniFrac distance of ASVs (Bray-Curtis) revealed no statistically significant clustering (p > 0.05) (data not shown).

At the phylum level, the intestinal microbiota composition of the study groups was similar at baseline. In the synbiotic group, the major phyla were Firmicutes (66.7%), Bacteroidetes (18.8%), Actinobacteria (7.6%), Proteobacteria (3.3%) and Verrucomicrobia (2.93%). In the synbiotic group, 12 weeks of intervention, at the phylum level, Firmicutes (66.0%), Bacteroidetes (24.0%), Actinobacteria (6.2%), Proteobacteria (2.0%) and Verrucomicrobia (1.22%) were observed. In the synbiotic group, the Bacteroidetes phylum was higher at 12 weeks of intervention than at baseline (24.0% vs. 18.8%, p < 0.01). In the placebo group, at baseline, the major phyla were Firmicutes (72.3%), Bacteroidetes (15.4%), Actinobacteria (8.7%), Proteobacteria (1.56%) and Verrucomicrobia (0.91%), and at 12 weeks of intervention, the major phyla were Firmicutes (69.2%), Bacteroidetes (22.6%), Actinobacteria (5.73%), Proteobacteria (1.8%) and Verrucomicrobia (0.59%). There was no difference between baseline and the 12th week of intervention in the placebo group (p > 0.05). There was also no difference between the synbiotic and placebo groups at the phylum level after 12 weeks of intervention (p > 0.05). In the synbiotic group, the Firmicutes/Bacteroidetes ratio was 3.54 at baseline and 2.75 at 12 weeks of intervention (p < 0.05). In the placebo group, the Firmicutes/Bacteroidetes ratio was 4.70 at baseline and 3.54 at 12 weeks of intervention (p < 0.05). After 12 weeks of intervention, the Firmicutes/Bacteroidetes ratio was also lower in the synbiotic group than in the placebo group (p < 0.05).

**Fig. 1:**
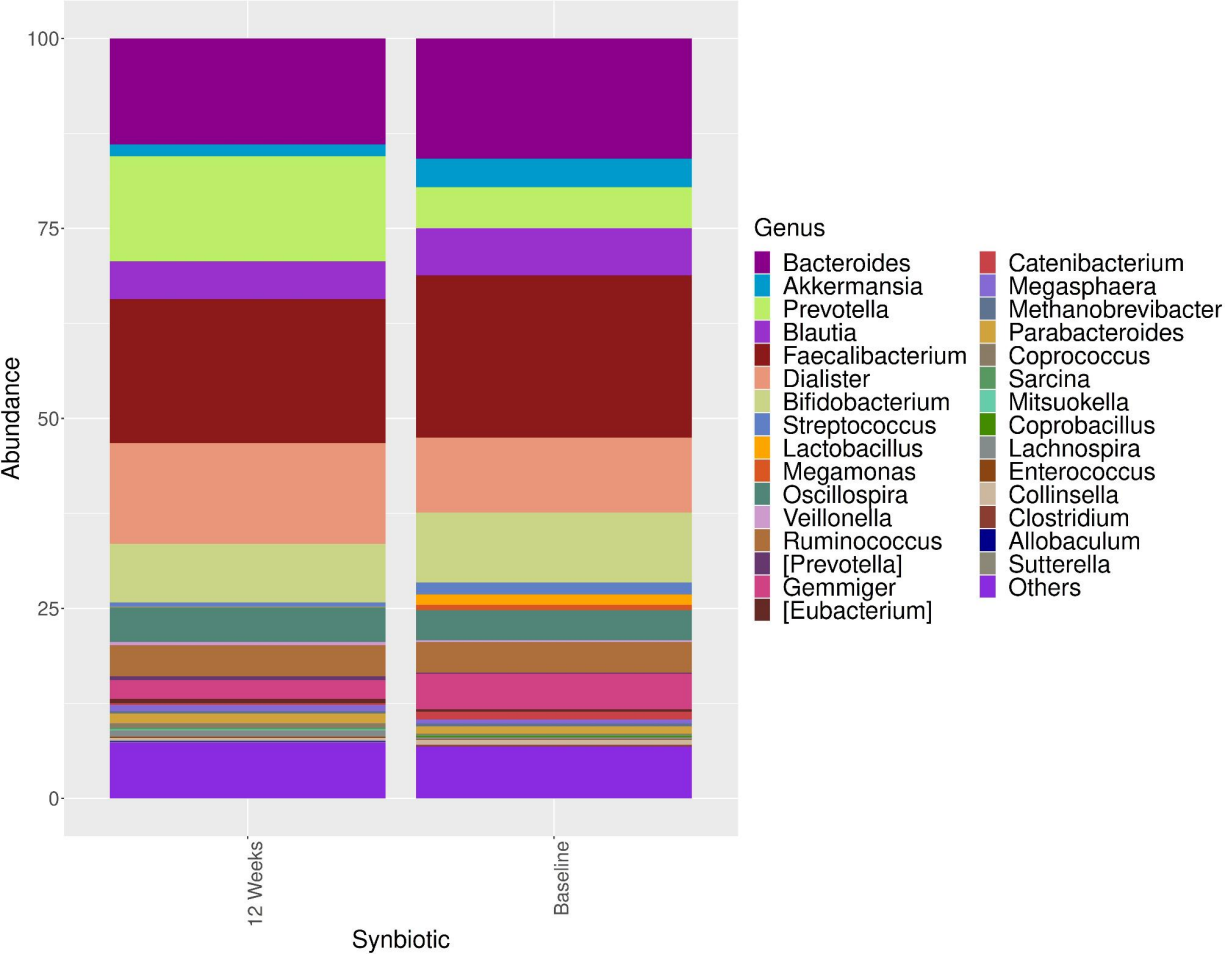
The distribution and comparison of the dominant microorganisms in the intestinal microbiota composition at baseline and at the 12th week of treatment in the synbiotic group at the genus level. Comparing the baseline, we observed a statistically significant increase in the genera *Prevotella* (5.28–14.4%, p < 0.001) and Dialister (9.68–13.4%; p < 0.05)

**Fig. 2:**
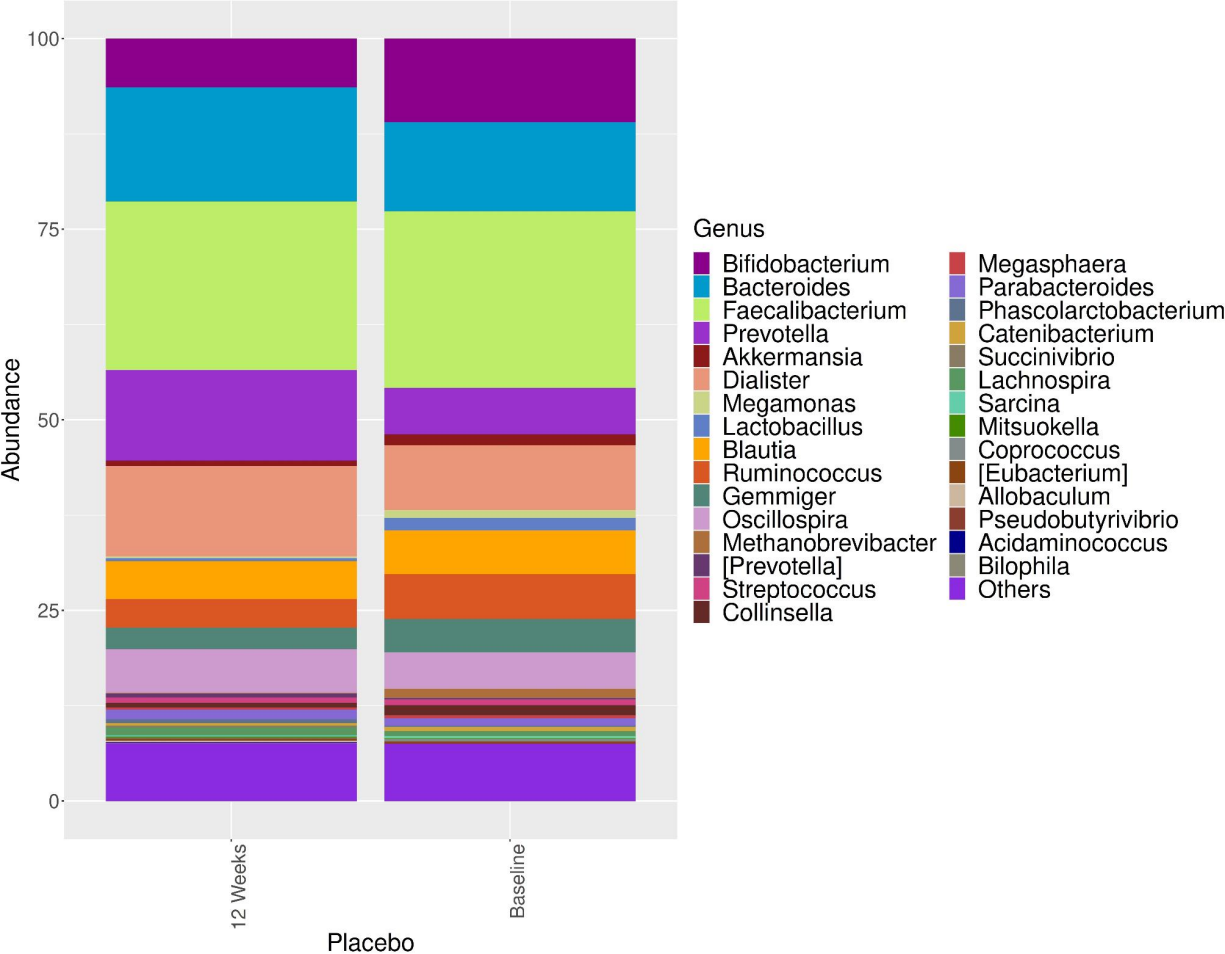
The distribution and comparison of the dominant microorganisms in the intestinal microbiota composition at baseline and at the 12th week of treatment in the placebo group at the genus level. Comparing the baseline, we observed a statistically significant increase in the genera *Prevotella* (6.4–12.4%, p < 0.01) and Oscillospira (4.95% vs. 5.70%, p < 0.001)

The genus level comparisons of the intestinal microbiota compositions of the synbiotic group and placebo group at baseline and at week 12 and among themselves at baseline and at week 12 are shown in Figs. 1 and 2.

In the synbiotic group, the most abundant genera were *Faecalibacterium* (20.5%), *Bacteroides* (16.3%), *Dialister* (9.68%), *Bifidobacterium* (9.55%), *Blautia* (6.62%), *Prevotella* (5.28%), *Gemmiger* (4.66%), *Akkermansia* (4.33%), *Ruminococcus* (4.14%), *Oscillospira* (3.91%), *Streptooccus* (2.27%), and *Lactobacillus* (1.76%). Twelve weeks of intervention, the most abundant genera were *Faecalibacterium* (18.7%), *Prevotella* (14.4%), *Bacteroides* (13.5%), *Dialister* (13.4%), *Bifidobacterium* (7.78%), *Blautia* (4.92%), *Oscillospira* (4.58%), *Ruminococcus* (4.03%), *Gemmiger* (2.52%), *Akkermansia* (1.77%), *Streptooccus* (1.01%), and *Lactobacillus* (0.37%) (Fig. 1). Comparing the baseline, we observed a statistically significant increase in the genera *Prevotella* (5.28–14.4%, p < 0.001) and *Dialister* (9.68–13.4%; p < 0.05) (Fig. 1).

In the placebo group, the most abundant genera were *Faecalibacterium* (23.2%), *Bacteroides* (11.4%), *Bifidobacterium* (10.9%), *Dialister* (8.72%), *Prevotella* (6.4%), *Ruminococcus* (6.07%), *Blautia* (5.74%), *Oscillospira* (4.95%), *Gemmiger* (4.6%), *Akkermansia* (4.33%), and *Lactobacillus* (2%). After 12 weeks of intervention, the most abundant genera were *Faecalibacterium* (22.0%), *Prevotella* (12.4%), *Bacteroides* (14.6%), *Dialister* (11.9%), *Bifidobacterium* (6.44%), *Blautia* (5.06%), *Oscillospira* (5.70%), *Ruminococcus* (3.77%), *Gemmiger* (3.02%), *Akkermansia* (0.92%), and *Lactobacillus* (0.76%). Comparing the baseline, we observed a statistically significant increase in the genera *Prevotella* (6.4–12.4%, p < 0.01) and *Oscillospira* (4.95% vs. 5.70%, p < 0.001) (Fig. 2).

**Fig. 3:**
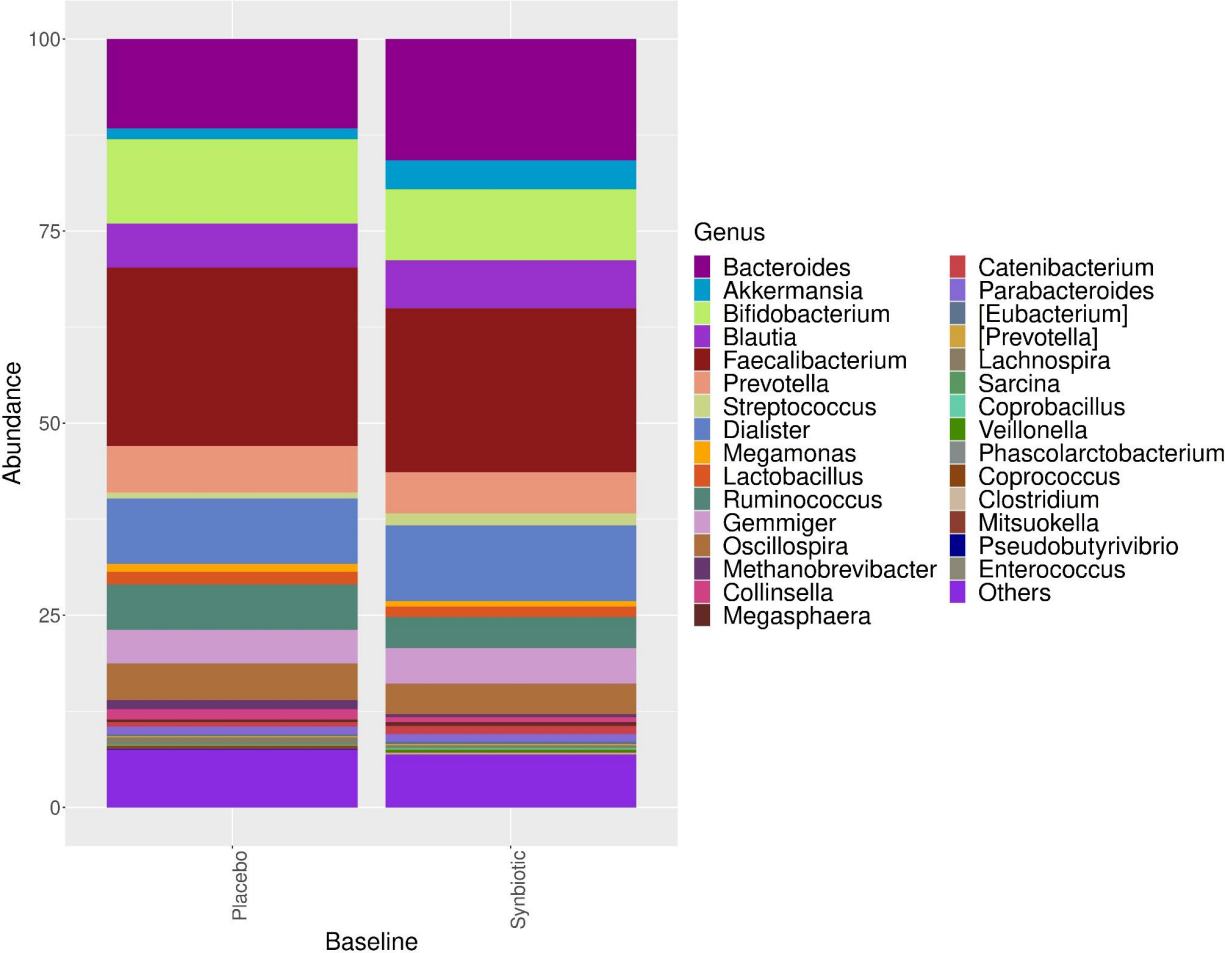
The distribution and comparison of the dominant microorganisms in the intestinal microbiota composition at baseline in the synbiotic and placebo groups at the genus level

**Fig. 4:**
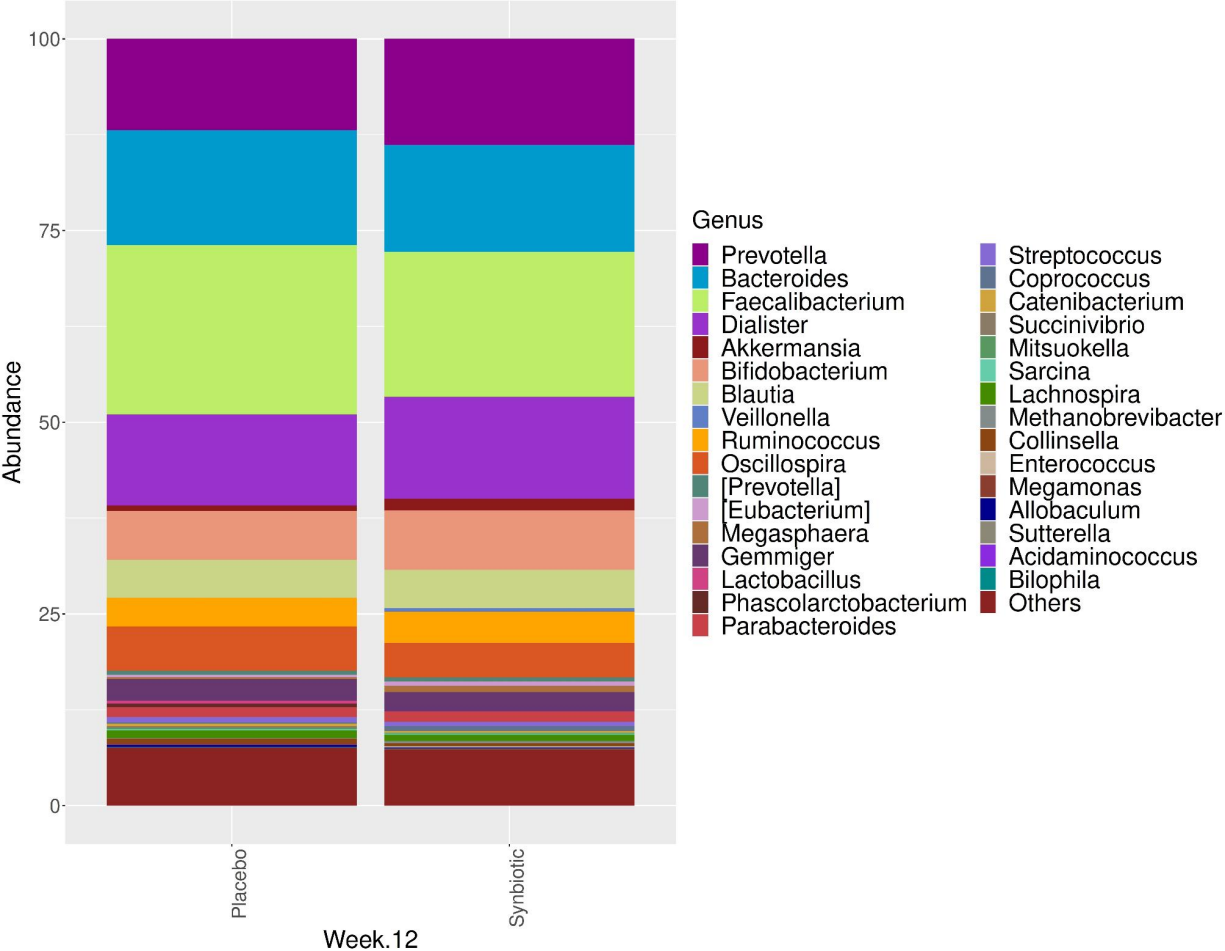
The distribution and comparison of the dominant microorganisms in the intestinal microbiota composition at the 12th week of treatment in the synbiotic and placebo groups at the genus level

At baseline and 12 weeks of intervention, there were no statistically significant differences in genera between the synbiotic and placebo groups (Figs. 3 and 4). *Faecalibacterium prausnitzii* is the most abundant strain in both groups at baseline and 12 weeks of intervention for synbiotic and placebo groups. There are no difference for the presence of *Faecalibacterium prausnitzii* at baseline and 12 weeks of intervention in the synbiotic group (35.6% and 32.9%, consecutively) and in the placebo group (23.2% and 22.0%) (p > 0.05).

**Fig. 5:**
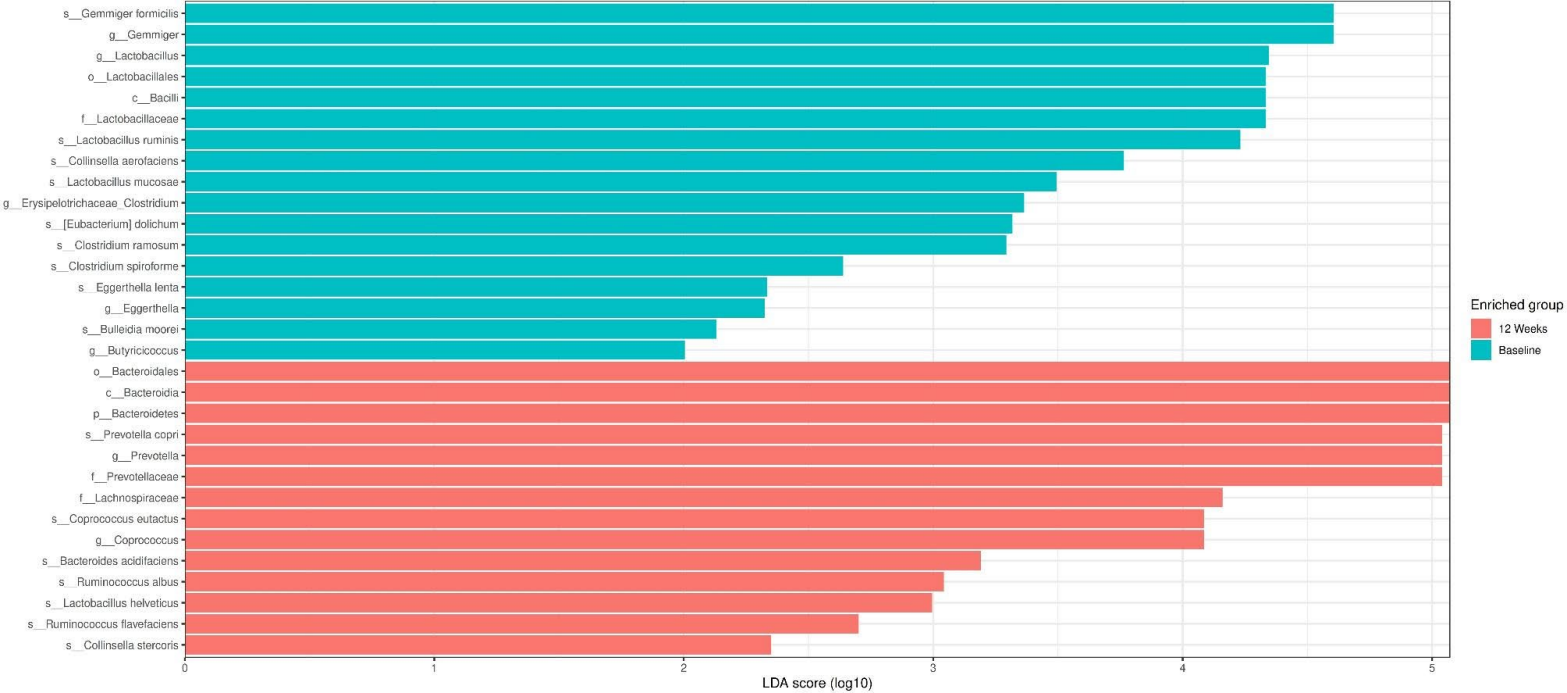
LEfSe analysis of stool samples at baseline and 3 months in the synbiotic group. Horizontal bars represent the log 10 converted LDA score, indicated by vertical dotted lines. Treatment initiation (green) 3 months (red). p—phylum, c-class, o—order; f—family, g—genus, s—species

Microbiota elements with an LDA score of > 2 were determined between the groups to show statistically significant taxonomies by LEFSe analysis in the study groups. At the beginning of the study, there was no significant difference between the synbiotic and placebo groups. In the placebo group, after 12 weeks of follow-up, an increase in the Bacteroidetes phylum, *Oscillospira* genus and *Oscillospira guillermondi* species was detected compared to the baseline period. In the synbiotic group, after 12 weeks of follow-up, an increase was detected in the Bacteroides phylum, Prevotella, Coprococcus genus and *Prevotella copri, Coprococcus eutactus, Ruminococcus albus, Ruminococcus flavefacines* species compared to the baseline period. In the synbiotic group, a decrease was detected in *Lactobacillus* and *Erysiplerotrichhaceae_Clostridium* genera and *Lactobacillus ruminis, Clostridium ramosum, Eubacterium dolichum, Clostridium spiroforme and Bulleidia moorei* species compared to the baseline period (Fig. 5).

At the end of the 12th week of the study, when the synbiotic and placebo groups were compared, *Bacteroides eggerthi* species were dominant in the placebo group, while *Collinsella stercoris* species were dominant in the synbiotic group.

## Discussion

Most of the studies on the effects of probiotics and synbiotics on obesity are related to anthropometric measurements, lipid parameters and non-alcoholic fatty liver disease, and there are few studies regarding their effects on intestinal microbiota composition especially In pediatric populations [8, 15–17, 30, 31]. This study is the first pediatric obesity study to show that 12 weeks of synbiotic supplementation results in positive changes in gastrointestinal microbiota composition in addition to improving BMI values.

In the present study, we observed a decrease in the Firmicutes/Bacteroidetes ratio in the synbiotic group after 12 weeks of intervention. Some studies have shown a significant reduction in Bacteroidetes and a higher Firmicutes to Bacteroidetes ratio in obese patients [16, 32]. An increase in the amount of Firmicutes to Bacteroidetes leads to methylation of obesity- and cardiovascular-related genes and influences the activity of hormones affecting metabolic function by increasing the ability to harvest energy [33]. Therefore, it seems that lowering the ratio of Firmicutes to Bacteroidetes is beneficial in managing obesity and obesity-related disorders. Previous studies have shown that the relative proportion of Bacteroidetes is decreased in obesity and that this proportion increases with weight loss [32, 34].

In patients with obesity, specific bacterial populations such as *Prevotellaceae, Blautia, Lactobacillus, Bifidobacterium spp*. were reported to be related to obesity as well. In our study, at baseline, in the synbiotic group, the major phyla were Firmicutes (66.7%), Bacteroidetes (18.8%), and Actinobacteria (7.6%), while they were Firmicutes (72.3%), Bacteroidetes (15.4%), and Actinobacteria (8.7%) in the placebo group (there were no differences between the groups). After 12 weeks of intervention, the Bacteroidetes phylum increased compared to baseline in the synbiotic group, while there was no change in the placebo group. Compared with the baseline, the genera *Prevotella* (5.28–14.4%) and *Dialister* (9.68–13.4%) increased significantly in the synbiotic group.

The synbiotic formulation used contains two lactobacilli (*L. acidophilus and L. rhamnosus*) and two bifidobacteria strains (*B. bifidum and B. longum*) and changes the intestinal microbiota composition. Previous limited studies conducted using *L. acidophilus and B. lactis* have found that these probiotic species can be associated with decreased body weight and body fat percentage [32]. A high-protein, low-carbohydrate, restricted-energy diet can be effectively used for weight loss in obese individuals. However, microbial breakdown of proteins within the large intestine has been associated with the production of genotoxic and cancer-associated metabolites [35]. Sergeev et al. [36] performed a placebo-controlled interventional trial designed to examine the effects of a combination of probiotic bacteria *L. acidophilus, B. lactis, B. longum, B. bifidum* and galactooligosaccharides on the intestinal microbiota in relation to changes in body composition and metabolic biomarkers in adult obese patients during weight loss intervention. This synbiotic combination resulted in a significant increase in the abundance of these probiotic genera in the gut after a 3-month intervention [36]. In addition, Prevotella and Gardnerella genera were significantly decreased after the synbiotic intervention. Contrary to this result, we observed increased Prevotella genera in the synbiotic as well as in the placebo group and an abundance of Prevotella copri in the synbiotic group. Special caution is warranted when analyzing the data referring to Prevotella, a complex genus linked both to health and disease and, possibly, influenced by race/ethnicity [36, 37]. In 2013, Larsen and colleagues [38] showed 12 that weeks of use of *L. salivarius Ls-33* might modify the fecal microbiota (significantly increased ratios of *Bacteroides, Prevotellae, Porphyromonas* group to *Firmicutes*-belonging bacteria, including *Clostridium cluster XIV, Blautia coccoides_ Eubacteria rectale group and Roseburia intestinalis*) in 50 obese adolescents.

Sergeev et al. [36] observed no differences in the community composition of gut microbiota between groups (synbiotic vs. placebo) and time points (end vs. beginning of trial) using parameters of alpha-diversity and beta-diversity [36]. In our study, there were no significant differences in alpha diversity indicators, including the Shannon index, between the synbiotic and placebo groups before the intervention. After 12 weeks of intervention, the observed ASVs and Chao 1 were lower in the synbiotic group than at baseline, while there was no difference in the placebo group or between the symbiotic and placebo groups. These results are compatible with a recent study that did not find a relationship between severe caloric restriction and changes in alpha diversity [39]. In humans, some studies have shown that obesity is associated with reduced bacterial diversity and an altered representation of bacterial species. Some studies have shown that bacterial diversity is significantly greater in subjects with obesity than in subjects without obesity [36]. Similar to Sergeev et al. [36], we speculated that the metabolic health benefits of synbiotics that we observed are likely not due to a direct influence of the interventions on species diversity.

In the synbiotic group, after 12 weeks of follow-up, an increase was detected in *Ruminococcus albus and Ruminococcus flavefacine* species, and a decrease was detected in *Eubacterium dolichum* species compared to the baseline period. The decrease in *Eubacterium dolichum* bacteria, which are frequently detected bacteria in patients with obesity, supports the positive effect of synbiotic application on microbiota. *Ruminococcus albus and Ruminococcus flavefacines* species are members of the Ruminococcus genus known to produce butyrate, which is a short-chain fatty acid that has some beneficial effects, including providing an energy source for colonocytes and acting as a histone deacetylase inhibitor, which has been linked to anticancer effects [36]. A relationship between human gut microbiota and metabolic disease exists, but what has to be clarified is whether the change in intestinal microbiota occurs before the development of inflammation or vice versa.

According to ISAAP, studies on a “synergistic synbiotic” that compare the synbiotic to the control can provide supportive evidence but do not constitute direct evidence that confirms a synergistic effect. Instead, a study including the combination, the substrate alone, the live microorganisms alone and a control should be conducted [11]. Hibberd et al. [40] aimed to investigate whether changes in the gut microbiota may be associated with the observed clinical benefits of probiotic *(Bifidobacterium animalis* subsp. *lactis* 42), prebiotic (Litesse Ultra polydextrose), synbiotic (*Bifidobacterium animalis* subsp. *lactis* 420 plus Litesse Ultra polydextrose) and placebo group. *Lactobacillus* and *Akkermansia* were more abundant in the probiotic alone group, while *Akkermansia, Christensenellaceae and Methanobrevibacter were increased in the symbiotic group*, while *Paraprevotella* was reduced. Increased *Christensenellaceae* was negatively correlated with the waist-hip ratio. Similar to our study, a two-arm parallel or crossover study would be sufficient to test a “complementary synbiotic”. As with all pro/synbiotics, the effect may vary depending on the strain identity, the number of colony forming units it contains, and the application time, and it should be kept in mind that the results obtained with one strain/preparation are not extrapolated for other strains. Jones et al. [41] evaluated 16 weeks of VSL#3 supplementation in 19 obese Hispanic adolescents and found that total adiposity and trunk adiposity had no significant effects on liver fat/fibrosis, insulin/glucose, gut microbial abundances or gut hormones.

The gut microbiota may participate in whole-body metabolism by affecting energy balance, glucose metabolism, and low-grade inflammation associated with obesity and related metabolic disorders. Many hypotheses have been proposed regarding the effect mechanisms of pre/pro/synbiotics on preventing weight gain or weight loss in obesity. These are reduction of inflammation, strengthening of intestinal epithelial barrier, prevention of bacterial translocation, modulation of intestinal enzyme activity, effects on neuroendocrine and immunological functions, inhibition of energy storage and food intake, reduction of dietary cholesterol absorption, prevention of reabsorption of bile acids in small intestines, and reduction of inflammation in intestines. The microbiota-obesity relationship is a complex process, and there are many factors that have not yet been clarified [8, 15]. The mechanism of action of probiotics and synbiotics on intestinal microbiota composition is strain-specific. In our study, the improvement in anthropometric measurements in the synbiotic group and the changes in the intestinal microbiota composition together show that the restoration of the microbiota should also be kept in mind in the mechanism of action.

Among the limitations of our study is that compliance with dietary intake and exercise recommendations was based on patient and parental reporting. Our patient’s compliance with the study products and study design was perfect at the beginning of the study; however, during the first year of the pandemic, the majority of the patients had no chance of coming to our clinic due to mitigation strategies (stay-at home orders or reorganization in the hospital). Our control group received same amounts of vitamins as the symbiotic group, and these vitamins might have an effect on intestinal microbiota composition while the anthropometric measurements were quite similar (except BMI values) at baseline and 12 weeks of intervention in placebo group. Symbiotic groups also received these vitamins if they have some beneficial effects on intestinal microbiota composition, and the end product of this symbiotic which is available in the market, includes symbiotics and vitamins. In addition, microbiota analyses included only bacteria, and other elements of the microbiota composition were not evaluated as well as short chain fatty acid levels. We enrolled children with expgenous obesity without comorbidities, and in the real world majority of the children and adults might have at least co-morbidties or complications. Results of this study are limited for patients with obesity with comorbidities.

## Conclusion

To the best of our knowledge, this trial was the first of its kind in the pediatric age to investigate the effect of synbiotic supplementation on anthropometric measurements and intestinal microbiota composition in obese children and adolescents. In our study, 12 weeks of synbiotic use was well tolerated and caused changes in microbiota composition. 12 weeks of synbiotic treatment was associated with both changes in microbiota composition and a decrease in average BMI; however, decreases in BMI were observed for the placebo group as well. Therefore, the differences in gut microbial community changes over time may be explained by synbiotic supplementation, though possibly through an interaction with BMI. Apart from our study, promising studies continue that new microbiota-targeted treatment approaches can also be used in the treatment of obesity. In addition, determining and preventing the factors that cause obesity with their effects on microbiota composition in the early period of life is an important strategy in obesity.

## Electronic supplementary material

Below is the link to the electronic supplementary material.

Supplementary Fig. 1: Flow chart of the study. Supplementary Fig. 2: Comparison of Chao 1 index at study baseline and 12 weeks in the synbiotic and placebo group. A statistical difference was found at the beginning of the study and at the end of the 12th week in the synbiotic group (p < 0.05). Supplementary Fig. 3: Shannon index comparison at study baseline and 12 weeks in the synbiotic and placebo groups. No statistical difference was found between the groups (p > 0.05). Supplementary Table 1: Anthropometric measurements and laboratory parameters of the synbiotic and placebo groups at the beginning of the study and at the end of the 12th week.

## Data Availability

The data that support the findings of this study are not openly available due to reasons of sensitivity and are available from the corresponding author upon reasonable request.

## Abbreviations

ASVs: Amplicon sequence variants
BMI: Body mass index
CDC: Centers for Disease Control and Prevention
CFU: Colony forming unit
ISAPP: International Scientific Association of Probiotics and Prebiotics
LEFSe: Linear discriminant analysis effect size
STORMS: Strengthening the Organization and Reporting of Microbiome Studies

## References

[1] -Ngowi EE, Wang YZ, Khattak S, Khan NH, Mahmoud SSM, Helmy YASH, Jiang QY, Li T, Duan SF, Ji XY, Wu DD (2021). Impact of the factors shaping gut microbiota on obesity. J Appl Microbiol.

[2] -Shanahan F, Ghosh TS, O’Toole PW (2021). The healthy microbiome-what is the definition of a healthy. Gut Microbiome? Gastroenterology.

[3] Hill JH, Round JL, SnapShot. Microbiota effects on host physiology (2021). Cell 184(10): 2796–6e1. 10.1016/j.cell.2021.04.026. 10.1016/j.cell.2021.04.026 33989551

[4] -Arumugam M, Raes J, Pelletier E, Le Paslier D, Yamada T, Mende DR (2011). Enterotypes of the human gut microbiome. Nature.

[5] -Selma-Royo M, Tarrazó M, García-Mantrana I, Gómez-Gallego C, Salminen S, Collado MC (2019). Shaping Microbiota during the First 1000 days of life. Adv Exp Med Biol.

[6] -Aires J (2021). First 1000 days of life: consequences of antibiotics on gut microbiota. Front Microbiol.

[7] -Ratsika A, Codagnone MC, O’Mahony S, Stanton C, Cryan JF (2021). Priming for life: early Life Nutrition and the Microbiota-Gut-Brain Axis. Nutrients.

[8] -Petraroli M, Castellone E, Patianna V, Esposito S (2021). Gut microbiota and obesity in adults and children: the state of the art. Front Pediatr.

[9] -Hill C, Guarner F, Reid G, Gibson GR, Merenstein DJ, Pot B, Morelli L, Canani RB, Flint HJ, Salminen S, Calder PC, Sanders ME (2014). Expert consensus document. The International Scientific Association for Probiotics and Prebiotics consensus statement on the scope and appropriate use of the term probiotic. Nat Rev Gastroenterol Hepatol.

[10] -Gibson GR, Hutkins R, Sanders ME, Prescott SL, Reimer RA, Salminen SJ, Scott K, Stanton C, Swanson KS, Cani PD, Verbeke K, Reid G (2017). Expert consensus document: the International Scientific Association for Probiotics and Prebiotics (ISAPP) consensus statement on the definition and scope of prebiotics. Nat Rev Gastroenterol Hepatol.

[11] -Swanson KS, Gibson GR, Hutkins R, Reimer RA, Reid G, Verbeke K, Scott KP, Holscher HD, Azad MB, Delzenne NM, Sanders ME (2020). The International Scientific Association for Probiotics and Prebiotics (ISAPP) consensus statement on the definition and scope of synbiotics. Nat Rev Gastroenterol Hepatol.

[12] -Marco ML, Sanders ME, Gänzle M, Arrieta MC, Cotter PD, De Vuyst L, Hill C, Holzapfel W, Lebeer S, Merenstein D, Reid G, Wolfe BE, Hutkins R (2021). The International Scientific Association for Probiotics and Prebiotics (ISAPP) consensus statement on fermented foods. Nat Rev Gastroenterol Hepatol.

[13] -Salminen S, Collado MC, Endo A, Hill C, Lebeer S, Quigley EMM, Sanders ME, Shamir R, Swann JR, Szajewska H, Vinderola G (2021). The International Scientific Association of Probiotics and Prebiotics (ISAPP) consensus statement on the definition and scope of postbiotics. Nat Rev Gastroenterol Hepatol.

[14] -Hruby A, Hu FB (2015). The epidemiology of obesity: a big picture. PharmacoEconomics.

[15] -Álvarez-Arraño V, Martín-Peláez S (2021). Effects of Probiotics and Synbiotics on Weight loss in subjects with overweight or obesity: a systematic review. Nutrients.

[16] -Perna S, Ilyas Z, Giacosa A, Gasparri C, Peroni G, Faliva MA, Rigon C, Naso M, Riva A, Petrangolini G, Redha A, Rondanelli A (2021). Is probiotic supplementation useful for the management of Body Weight and other anthropometric measures in adults affected by overweight and obesity with metabolic related Diseases? A systematic review and Meta-analysis. Nutrients.

[17] -Mohammadi H, Ghavami A, Hadi A, Askari G, Symonds M, Miraghajani M (2019). Effects of pro-/synbiotic supplementation on anthropometric and metabolic indices in overweight or obese children and adolescents: a systematic review and meta-analysis. Complement Ther Med.

[18] -Ipar N, Aydogdu SD, Yildirim GK, Inal M, Gies I, Vandenplas Y, Dinleyici EC (2015). Effects of synbiotic on anthropometry, lipid profile and oxidative stress in obese children. Benef Microbes.

[19] -Kilic Yildirim G, Dinleyici M, Vandenplas Y, Dinleyici EC (2022). Effects of Multispecies Synbiotic supplementation on anthropometric measurements, glucose and lipid parameters in children with exogenous obesity: a Randomized, double blind, placebo-controlled clinical trial (Probesity-2 trial). Front Nutr.

[20] -Mirzayi C, Renson A, Genomic S (2021). Reporting guidelines for human microbiome research: the STORMS checklist. Nat Med.

[21] Defining childhood weight status. https://www.cdc.gov/obesity/childhood/defining.html. Last Access Date January 20, 2022.

[22] -Bolyen E, Rideout JR, Dillon MR, Bokulich NA, Abnet CC, Al-Ghalith GA, Alexander H (2019). Reproducible, interactive, scalable and extensible microbiome data science using QIIME 2. Nat Biotechnol.

[23] -Callahan BJ, McMurdie PJ, Rosen MJ, Han AW, Johnson AJ, Holmes SP (2016). DADA2: high-resolution sample inference from Illumina amplicon data. Nat Methods.

[24] -Schloss PD (2021). Amplicon sequence variants artificially Split bacterial genomes into separate clusters. mSphere.

[25] -Werner JJ, Koren O, Hugenholtz P, DeSantis TZ, Walters WA, Caporaso JG, Angenent LT, Knight R, Ley RE (2012). Impact of training sets on classification of high-throughput bacterial 16s rRNA gene surveys. Isme J.

[26] -McMurdie PJ, Holmes S (2013). Phyloseq: an R package for reproducible interactive analysis and graphics of microbiome census data. PLoS ONE.

[27] R Core Team (2021). R: a language and environment for statistical computing.

[28] -Love MI, Huber W, Anders S (2014). Moderated estimation of fold change and dispersion for RNA-seq data with DESeq2. Genome Biol.

[29] -Segata N, Izard J, Waldron L, Gevers D, Miropolsky L, Garrett WS, Huttenhower C (2011). Metagenomic biomarker discovery and explanation. Genome Biol.

[30] -Kong XJ, Wan G, Tian R, Liu S, Liu K, Clairmont C, Lin X, Zhang X, Sherman H, Zhu J, Wang Y, Fong M, Li A, Wang BK, Wang J, Liu J, Yu Z, Shen C, Cui X, Cao H, Du T, Cao X (2021). The Effects of Probiotic supplementation on Anthropometric Growth and Gut Microbiota composition in patients with Prader-Willi Syndrome: a Randomized double-blinded placebo-controlled trial. Front Nutr.

[31] -Verma A, Nelson MT, DePaolo WR, Hampe C, Roth CL (2021). A randomized double-blind placebo controlled pilot study of probiotics in adolescents with severe obesity. J Diabetes Metab Disord.

[32] Magne F, Gotteland M, Gauthier L, Zazueta A, Pesoa S, Navarrete P, Balamurugan R (2020). The Firmicutes/Bacteroidetes ratio: a relevant marker of gut dysbiosis in obese patients?. Nutrients.

[33] -Daniali M, Nikfar S, Abdollahi M (2020). A brief overview on the use of probiotics to treat overweight and obese patients. Expert Rev Endocrinol Metab.

[34] -Kassaian N, Feizi A, Rostami S, Aminorroaya A, Yaran M, Amini M (2020). The effects of 6 mo of supplementation with probiotics and synbiotics on gut microbiota in the adults with prediabetes: a double blind randomized clinical trial. Nutrition.

[35] -Al Hinai EA, Kullamethee P, Rowland IR, Swann J, Walton GE, Commane DM (2019). Modelling the role of microbial p-cresol in colorectal genotoxicity. Gut Microbes.

[36] -Sergeev IN, Aljutaily T, Walton G, Huarte E (2020). Effects of Synbiotic supplement on human gut microbiota, body composition and weight loss in obesity. Nutrients.

[37] -Stanislawski MA, Dabelea D, Lange LA, Wagner BD, Lozupone CA (2019). Gut microbiota phenotypes of obesity. NPJ Biofilms Microbiomes.

[38] Larsen N, Vogensen FK, Gøbel RJ, Michaelsen KF, Forssten SD, Lahtinen SJ, Jakobsen M. (2013) Effect of Lactobacillus salivarius Ls-33 on fecal microbiota in obese adolescents. Clin Nutr 32(6): 935 – 40. 940. 10.1016/j.clnu.2013.02.007. 10.1016/j.clnu.2013.02.007 23510724

[39] -Ott B, Skurk T, Hastreiter L, Lagkouvardos I, Fischer S, Büttner J, Kellerer T, Clavel T, Rychlik M, Haller D, Hauner H (2017). Effect of caloric restriction on gut permeability, inflammation markers, and fecal microbiota in obese women. Sci Rep.

[40] -Hibberd AA, Yde CC, Ziegler ML, Honoré AH, Saarinen MT, Lahtinen S, Stahl B, Jensen HM, Stenman LK (2019). Probiotic or synbiotic alters the gut microbiota and metabolism in a randomised controlled trial of weight management in overweight adults. Benef Microbes.

[41] -Jones RB, Alderete TL, Martin AA, Geary BA, Hwang DH, Palmer SL, Goran MI (2018). Probiotic supplementation increases obesity with no detectable effects on liver fat or gut microbiota in obese hispanic adolescents: a 16-week, randomized, placebo-controlled trial. Pediatr Obes.

