# Supplementary material for: Effects of synbiotic supplementation on intestinal microbiota composition in children and adolescents with exogenous obesity: (Probesity-2 trial)
Source: Gut Pathog. 2023 Jul 21;15:36. doi: 10.1186/s13099-023-00563-y (PMC10360342; doi:10.1186/s13099-023-00563-y)
Supplement: Supplementary file 1 — Supplementary Fig. 1: Flow chart of the study. Supplementary Fig. 2: Comparison of Chao 1 index at study baseline and 12 weeks in the synbiotic and placebo group. A statistical difference was found at the beginning of the study and at the end of the 12th week in the synbiotic group (p < 0.05). Supplementary Fig. 3: Shannon index comparison at study baseline and 12 weeks in the synbiotic and placebo groups. No statistical difference was found between the groups (p > 0.05). Supplementary Table 1: Anthropometric measurements and laboratory parameters of the synbiotic and placebo groups at the beginning of the study and at the end of the 12th week. [file 13099_2023_563_MOESM1_ESM.docx]

**Supplementary Figure 1.** Flow chart of the study.

**
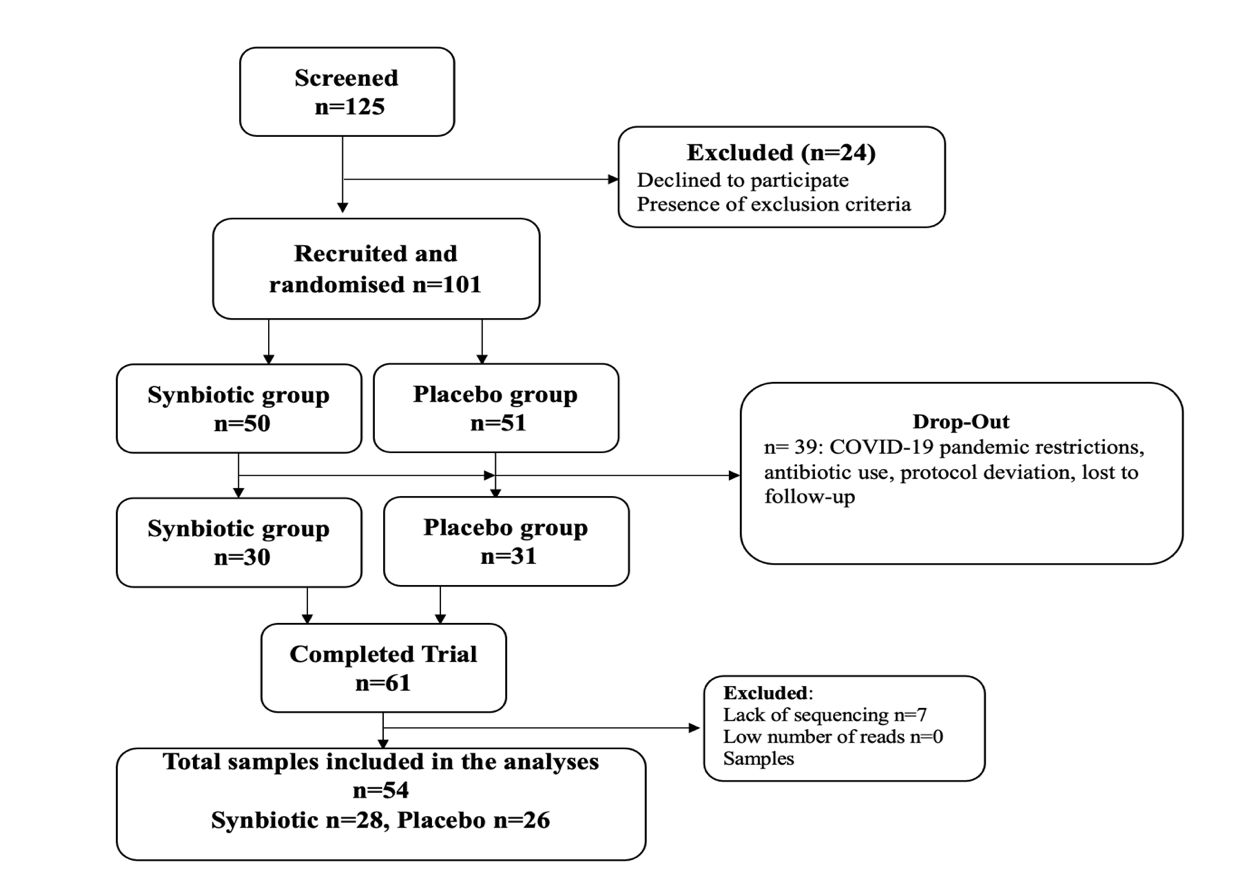
**


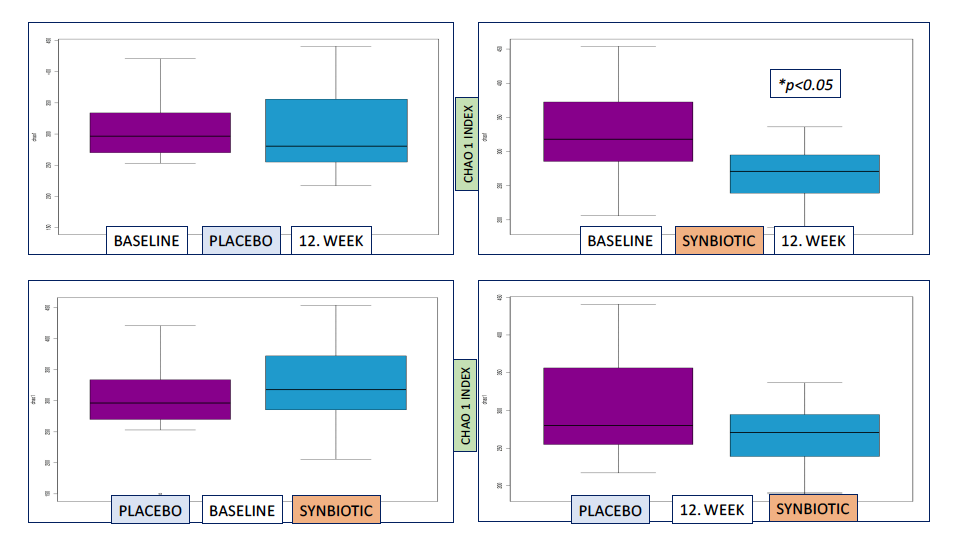


**Supplementary Figure 2.** Comparison of Chao 1 index at study baseline and 12 weeks in the synbiotic and placebo group. A statistical difference was found at the beginning of the study and at the end of the 12th week in the synbiotic group (p<0.05)


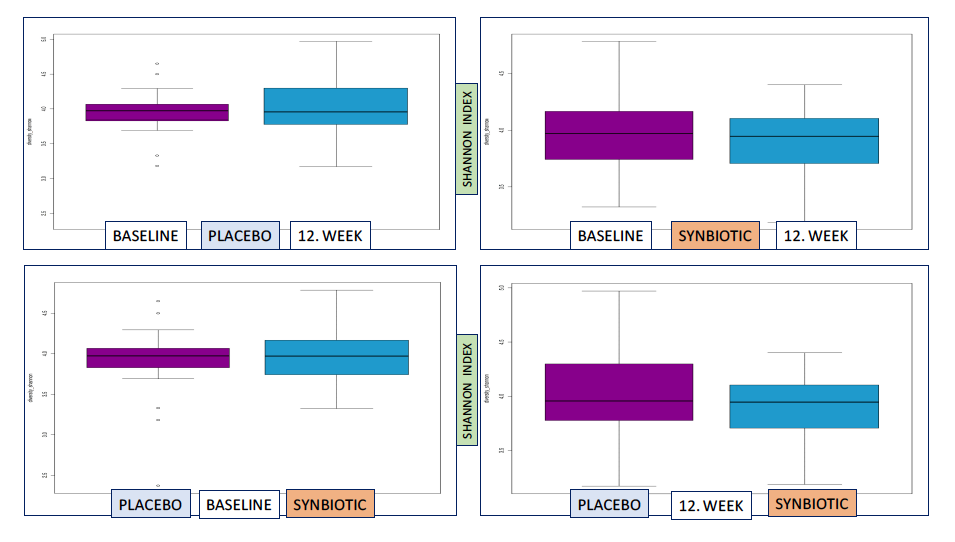


**Supplementary Figure 3.** Shannon index comparison at study baseline and 12 weeks in the synbiotic and placebo groups. No statistical difference was found between the groups (p>0.05)

**Supplementary Table 1. Anthropometric measurements and laboratory parameters of the synbiotic and placebo groups at the beginning of the study and at the end of the 12th week**

|  | **Synbiotic Group**  **n=28** | | | **Placebo Group**  **n=26** | | |
| --- | --- | --- | --- | --- | --- | --- |
| **Age (months)** | 137 ± 36 | | | 144 ± 31 | | |
|  | **Baseline** | **12^th^ weeks** | **p1** | **Baseline** | **12^th^ weeks** | **p2** |
| **Weight (kg)** | 66.3 ± 19.4 | 63.9 ± 19.6 | ***p<0.001*** | 71.7 ± 22.2 | 70.8 ± 20.9 | ns |
| **Body mass index (kg/m^2^)** | 28.3 ± 4.0 | 26.9 ± 4.31 | ***p<0.001*** | 29.2±6.25 | 28.4 ± 5.84 | **p<0.05** |
| **Total cholesterol (mg/dl)** | 160.3 ± 26.7 | 163.3 ± 29.8 | ns | 156.4 ± 36.1 | 157.4 ± 35.0 | ns |
| **HDL-C (mg/dl)** | 43.6 ± 7.3 | 43.6 ± 7.0 | ns | 43.5 ± 11.7 | 43.9 ± 10.3 | ns |
| **LDL-C (mg/dl)** | 106.7 ± 27.3 | 109.4 ± 29.0 | ns | 99.8 ± 33.6 | 101.6 ± 34.2 | ns |
| **Triglyceride (mg/dl)** | 126.8 ± 48.8 | 121.7 ± 63.5 | ns | 142.6±49.0 | 141.4±61.8 | ns |
| **ALT (IU/L)** | 27.3 ± 19.6 | 27.9±28.6 | ns | 22.1±12.3 | 18.6±5.24 | ns |
| **HOMA-IR** | 4.45 ± 2.67 | 3.80 ± 1.82 | ns | 4.19 ± 2.83 | 3.51 ± 1.80 | ns |

**The data were expressed as mean ± standart deviation. p1: Baseline vs. 12th week in synbiotic group, p2, Baseline vs. 12th week in placebo group; BMI (body mass index), ALT, alanine aminotransferase; HDL-C, high-density lipoprotein-cholesterol; LDL-C, low-density lipoprotein-cholesterol; HOMA-IR, homoeostatic model assessment for insulin resistance; Bold values are statistically significant**
